# Implicit and Explicit Number-Space Associations Differentially Relate to Interference Control in Young Adults With ADHD

**DOI:** 10.3389/fpsyg.2018.00775

**Published:** 2018-05-24

**Authors:** Carrie Georges, Danielle Hoffmann, Christine Schiltz

**Affiliations:** ^1^Institute of Cognitive Science and Assessment, Research Unit Education, Culture, Cognition and Society, Faculty of Language and Literature, Humanities, Arts and Education, University of Luxembourg, Luxembourg, Luxembourg; ^2^Luxembourg Centre for Educational Testing, Faculty of Language and Literature, Humanities, Arts and Education, University of Luxembourg, Luxembourg, Luxembourg

**Keywords:** SNARC effect, magnitude processing, interference control, Stroop effect, Flanker effect, individual differences

## Abstract

Behavioral evidence for the link between numerical and spatial representations comes from the spatial-numerical association of response codes (SNARC) effect, consisting in faster reaction times to small/large numbers with the left/right hand respectively. The SNARC effect is, however, characterized by considerable intra- and inter-individual variability. It depends not only on the explicit or implicit nature of the numerical task, but also relates to interference control. To determine whether the prevalence of the latter relation in the elderly could be ascribed to younger individuals’ ceiling performances on executive control tasks, we determined whether the SNARC effect related to Stroop and/or Flanker effects in 26 young adults with ADHD. We observed a divergent pattern of correlation depending on the type of numerical task used to assess the SNARC effect and the type of interference control measure involved in number-space associations. Namely, stronger number-space associations during parity judgments involving implicit magnitude processing related to weaker interference control in the Stroop but not Flanker task. Conversely, stronger number-space associations during explicit magnitude classifications tended to be associated with better interference control in the Flanker but not Stroop paradigm. The association of stronger parity and magnitude SNARC effects with weaker and better interference control respectively indicates that different mechanisms underlie these relations. Activation of the magnitude-associated spatial code is irrelevant and potentially interferes with parity judgments, but in contrast assists explicit magnitude classifications. Altogether, the present study confirms the contribution of interference control to number-space associations also in young adults. It suggests that magnitude-associated spatial codes in implicit and explicit tasks are monitored by different interference control mechanisms, thereby explaining task-related intra-individual differences in number-space associations.

## Introduction

Numbers and space are closely associated in the human mind (e.g., Dehaene and Brannon, 2011). The most extensively studied and replicated behavioral evidence for this association is without a doubt the spatial-numerical association of response codes (SNARC) effect (Dehaene et al., 1993). It describes the observation that individuals from Western societies are typically faster on their left/right hand-side for relatively small/large numbers respectively, when doing binary classifications on numbers. The SNARC effect was first documented in an experiment where numerical magnitude information was task-relevant (termed “the magnitude SNARC effect”) in that individuals judged whether a centrally displayed number was smaller or larger than a given standard (Dehaene et al., 1990). Subsequent experiments, however, demonstrated that numerical magnitude does not need to be task-relevant to observe the SNARC effect, since it was also evidenced during parity judgments (termed “the parity SNARC effect”; e.g., Dehaene et al., 1993).

Three spatial coding mechanisms were proposed to account for spatial-numerical interactions, including a visuospatial, verbal-spatial, and working memory (WM) account (for a review, see e.g., Fischer and Shaki, 2014). According to the dominant and most traditional *visuospatial* account, numbers are mentally represented along a continuous left-to-right-oriented spatial representational medium, also known as the mental number line (MNL), with small/large numbers located on its left/right respectively, at least in Western societies (Moyer and Landauer, 1967; Restle, 1970; Dehaene et al., 1993). An alternative view suggests that number-space associations arise from categorical *verbal-spatial* coding. The latter account is based on the polarity correspondence principle by Proctor and Cho (2006) and assumes that the SNARC effect results from the polar correspondence between the verbal categorical concepts “small” and “left” (both assigned to the same polarity) as well as “large” and “right” (both assigned to the opposing polarity). A final explanation for the link between numbers and space was provided by Fias et al. (2011), who argued that spatial-numerical interactions are task-specific associations established within *WM* (see also van Dijck and Fias, 2011; Abrahamse et al., 2016; Fias and van Dijck, 2016). More concretely, task-relevant numerical magnitudes are temporarily activated in their canonical order within a horizontal left-to-right oriented spatial sequence in WM. Spatial-numerical interactions then result from internal shifts of spatial attention within this encoded numerical sequence, with positions from the beginning/end of the sequence eliciting faster left-/right-sided responses respectively.

### Inter-Individual Differences in Number-Space Associations

The strength of number-space associations considerably varies between individuals. For instance, variability is explained by inter-individual differences in mathematical skills. Participants with lower arithmetic performances featured stronger number-space associations in the parity judgment task (e.g., Georges et al., 2017; but see Cipora and Nuerk, 2013). Similarly, more pronounced parity SNARC effects were observed in humanities students with than without math difficulties (Hoffmann et al., 2014a), while the weakest number-space associations were evidenced in math professionals (Cipora et al., 2016). The parity SNARC effect also depends on math anxiety, with more anxious individuals displaying stronger number-space associations (Georges et al., 2016). Furthermore, it was shown to increase with age (Hoffmann et al., 2014b; Ninaus et al., 2017).

In addition to this, inter-individual variability in the parity SNARC effect has recently been shown to relate to differences in inhibitory control as indexed by the Stroop effect (Hoffmann et al., 2014b; for a review on the Stroop effect, see MacLeod, 1991; see also Stroop, 1935). Participants with weaker interference control in the Stroop paradigm featured stronger number-space associations in the parity judgment task. The relation between number-space associations during parity judgments and inhibitory control might be explained by the need to inhibit numerical magnitude and its associated spatial code to accurately respond based on the number’s parity status. It should, however, be noted that the relation between weaker interference control in the Stroop task and stronger parity SNARC effects was most pronounced in the elderly. It did not reach significance in young healthy individuals, which the authors ascribed to their near ceiling performances on the Stroop task.

### Intra-Individual Differences in Number-Space Associations

Apart from inter-individual differences in the SNARC effect, number-space associations also vary intra-individually depending on the number processing task. For instance, Georges et al. (2017) observed no significant relation between the SNARC effects in a parity judgment and magnitude classification task (at least at the sample level – positive and negative correlations were evidenced in individuals with object and spatial visualization styles respectively). Moreover, verbal and visuospatial WM load selectively abolished the parity and magnitude SNARC effects respectively (Herrera et al., 2008; van Dijck et al., 2009). In addition, hemi-neglect patients were shown to display regular number-space associations in the parity judgment task, where access to numerical magnitude is implicit, but featured an atypical SNARC effect in the explicit magnitude classification task (Priftis et al., 2006; Zorzi et al., 2012). The SNARC effects in implicit and explicit tasks were also shown to associate with different cognitive factors. Namely, only the magnitude SNARC effect related to inter-individual differences in visualization cognitive styles (Georges et al., 2017; see also Kozhevnikov et al., 2005; Chabris et al., 2006). Furthermore, the relation between weaker arithmetic performances and stronger SNARC effects during parity judgments (e.g., Hoffmann et al., 2014a; Georges et al., 2017; but see Cipora and Nuerk, 2013) was not observed for number-space associations in the explicit magnitude classification task. Altogether, these findings suggest that numbers might be associated with qualitatively different spatial codes depending on the implicit or explicit nature of the numerical processing task.

### Interference Control and Inter-/Intra-Individual Differences in Number-Space Associations

The present study aimed to (a) replicate the previously reported relationship between implicit number-space associations and inhibitory control (Hoffmann et al., 2014b) and (b) investigate whether this relationship extends to explicit magnitude processing.

While the “parity SNARC-Stroop” relation was significant in a group composed of young and elderly healthy participants, it was mainly driven by the elderly and did not reach significance in the young subgroup (Hoffmann et al., 2014b). We reasoned that this result pattern might be caused by the fact that young healthy adults achieved near ceiling performances on the Stroop task. In the current study, we therefore focussed on young individuals featuring atypical inhibitory control and included only participants formally diagnosed with ADHD and/or displaying symptoms consistent with ADHD according to the Adult ADHD Self-Report Scale (Kessler et al., 2005). These people not only feature weaker interference control (Walker et al., 2000; Rapport et al., 2001; Lansbergen et al., 2007), but their deficits are also highly variable (Lovejoy et al., 1999; Sergeant et al., 2002; Seidman, 2006). Such inter-individual variability in inhibitory control deficits should increase the statistical power of detecting significant relations with other continuous variables (e.g., Goodwin and Leech, 2006). This enables us to verify whether the previously reported null relation between the parity SNARC and Stroop effects in the younger healthy individuals (Hoffmann et al., 2014b) can indeed be ascribed to their near ceiling performances on the Stroop task. Finding evidence for a significant association between number-space associations in the parity judgment task and interference control in the Stroop paradigm in a relatively younger population would considerably strengthen the critical involvement of inhibitory control mechanisms in the spatial coding processes underlying the parity SNARC effect.

In addition to interference control in the Stroop task, the present study also determined whether executive control processes in the arrowhead version of the Flanker task (e.g., Stins et al., 2004; Davelaar and Stevens, 2009; for the original version, see Eriksen and Eriksen, 1974) might relate to inter-individual differences in number-space associations during parity judgments. Even though conflict occurs in both the Stroop and Flanker paradigms, its nature and processing likely differ depending on the executive control task. For instance, while elderly people were shown to display weaker interference control in the Stroop task than young adults (West and Alain, 2000; Van der Elst et al., 2006), inhibitory control in the Flanker task did not differ between younger and older participants (Falkenstein et al., 2001; Nieuwenhuis et al., 2002). Moreover, heritability of interference control was evidenced in the Stroop but not the Flanker task (Stins et al., 2004). In addition, interference control in the Stroop but not the Flanker task was related to WM capacity (Stins et al., 2005). Furthermore, relations could be evidenced neither between the time needed for conflict resolution nor between the interference scores in the Stroop and Flanker tasks (Stins et al., 2005). Conflict processing in the Flanker task was shown to relate to the activation of the right dorsolateral prefrontal cortex and the insula (Zhu et al., 2010; Zmigrod et al., 2016). Conversely, neural responses reflecting the Stroop effect were measured in a broader network including not only the right dorsolateral prefrontal cortex, but also the posterior parietal, anterior cingulate and left premotor cortices (van Veen and Carter, 2005; Melcher and Gruber, 2009; Kim et al., 2011; for a meta-analysis, see Nee et al., 2007). These findings thus suggest that Stroop and Flanker effects likely reflect qualitatively different executive control processes. Consequently, contrasting their relations with number-space associations will allow for a better understanding of the specific inhibitory control mechanisms contributing to spatial-numerical interactions.

In a second step, we aimed to assess the relations between the SNARC effect during explicit magnitude classifications and inhibitory control indexed by the Stroop and Flanker effects, since number-space associations were previously shown to vary intra-individually depending on the implicit or explicit nature of the number processing task (van Dijck et al., 2009; Georges et al., 2017). This will inform us about the involvement of inhibitory control processes in the spatial coding processes underlying the magnitude SNARC effect and as such their role in intra-individual differences in number-space associations.

## Materials and Methods

This study was reviewed and approved by the Ethics Review Panel (ERP) of the University of Luxembourg. All participants gave written informed consent and received a small monetary reward for their participation.

### Participants

The study was advertised to the university students via their email addresses. Students could take part in the study if they were formally diagnosed with ADHD (Attention-Deficit/Hyperactivity Disorder) and/or if they considered themselves as being easily distracted and unable to concentrate. A total of 42 students signed up for the study, of which 5 had a formal diagnosis of ADHD. Participants had various backgrounds with different mother tongues (e.g., English, Finnish, French, German, Greek, Russian, Spanish, etc.) and their study fields ranged from mathematics and physics over law to humanities. None of the participants suffered from any comorbid learning disabilities such as dyslexia or dyscalculia.

### Procedure and Tasks

Before the start of the experiment, the 42 students that had signed up for the study completed the 6-item version of the World Health Organization Adult ADHD Self-Report Scale V 1.1 (ASRS) symptom checklist (Kessler et al., 2005; for psychometric properties, see Adler et al., 2006; Matza et al., 2011). This was to ensure that individuals not formally diagnosed with ADHD displayed symptoms consistent with this disorder. Participants without a formal diagnosis of ADHD that did not feature ADHD traits according to this self-report scale were excluded prior to the start of the study. This reduced the study sample to a total of 35 participants.

These participants completed the experimental tasks during two testing sessions that were run on separate days with an upper limit of 1 week apart. Following standard practice in individual differences research (e.g., Carlson and Moses, 2001), all participants performed the tests in the same order and trial sequences were identical for all participants in every task. On the first testing day, participants completed the speeded matching-to-sample task, the parity judgment task, the magnitude classification task and the Flanker task. These computerized tasks were programmed in E-prime (Version 1.2 or 2.0.8.79) and administered on a Windows computer. The classical verbal paper-and-pencil version of the Stroop task was implemented on the second testing day.

Prior to data analysis, 4 students were excluded from the sample since they did not complete all the tests. After removal of these participants, outliers were identified for each of the measures described below. A total of 5 participants had to be removed, since their performances fell 2.5 standard deviations (*SD*) below or above the mean group performances on at least one of the measures. All statistical analyses were thus conducted on data obtained from 26 individuals.

#### Parity Judgment and Magnitude Classification Tasks

**FIGURE 1:**
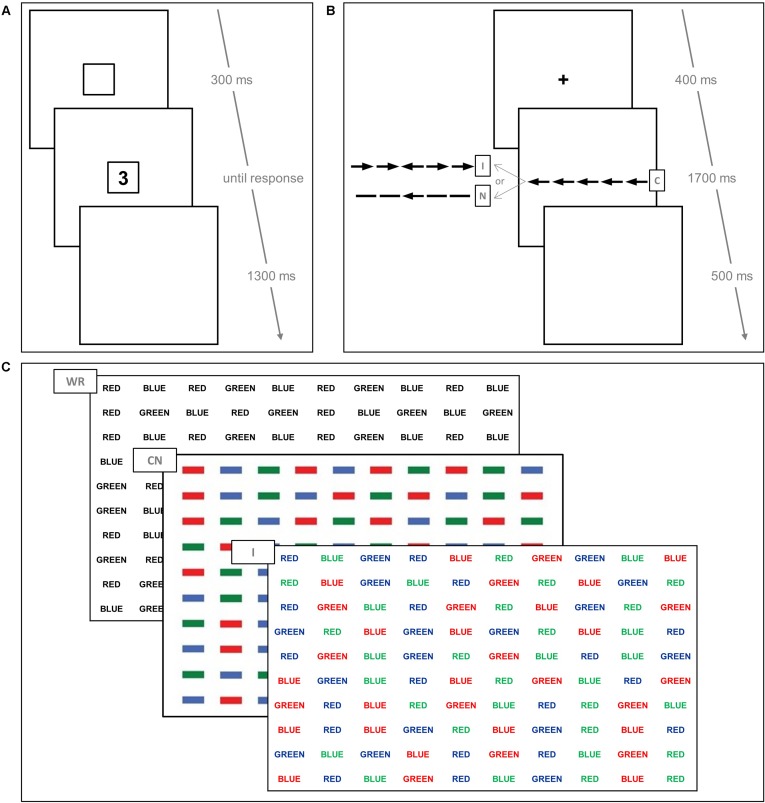
Schematic representation of the different experimental tasks. Trial sequence in the computerized parity judgment and magnitude classification tasks **(A)**. Trial sequence with a congruent target stimulus (C) in the computerized Flanker task **(B)**. Incongruent (I) and neutral (N) target stimuli are displayed on the left **(B)**. Word reading (WR), color naming (CN) and interference (I) conditions in the classical 100-item verbal paper-and-pencil version of the Stroop task **(C)**.

The ***parity judgment task*** (adapted from Dehaene et al., 1993; see also Georges, 2017; see **Figure 1A**) was administered to determine number-space associations in a task with ***implicit numerical magnitude processing***. The experiment consisted of 288 experimental trials divided equally across two blocks. Each experimental trial started with an empty black-bordered square (6.87° × 6.87°) on a white background. After 300 ms, one of eight possible stimuli (Arabic digits: 1, 2, 3, 4, 6, 7, 8, or 9; color: black; font: Arial; point size: 64) appeared in the center of the black-bordered square and remained until response. The inter-trial interval consisted of a blank screen of 1300 ms. In the first block, participants judged as quickly as possible whether the presented number was odd/even by pressing the “A”/“L” key on a QWERTZ keyboard respectively. This stimulus-response mapping was reversed for all participants in the second block. Each target number was displayed 18 times per block. The sequence in which the target stimuli appeared was pseudo-randomized in a way that no target number could appear twice in a row, and the correct response could not be on the same side more than three times consecutively. Each block started with 12–20 training trials, depending on response accuracy. Participants were given a small break half-way through each block.

The ***magnitude classification task*** (adapted from Bull et al., 2005; van Galen and Reitsma, 2008; see also Georges, 2017; see **Figure 1A**) was administered to determine number-space associations in a task with ***explicit numerical magnitude processing***. The experiment was identical to the parity judgment task with the exception that it only consisted of 144^1^ trials and that participants had to judge whether the centrally presented single Arabic number was smaller/larger than five by pressing the “A”/“L” key respectively in the first block. This stimulus-response mapping was again reversed for all participants in the second block.

Data from the training sessions was not analyzed (for comparable data analysis, see Georges et al., 2017). The mean error rate on experimental trials was 2.52 and 2.56% in the parity judgment and magnitude classification task respectively [*F*(1,25) = 0.006; *p* = 0.94; $\eta_{p}^{2}$ = 0.00]. Errors were not further analyzed. Reaction times (RTs) shorter or longer than 2.5 *SD* from the individual mean were considered as outliers and discarded prior to data analysis (2.86 and 3.19% of all correct trials in the parity judgment and magnitude classification task respectively, *F*(1,25) = 1.55; *p* = 0.23; $\eta_{p}^{2}$ = 0.06).

SNARC effect regression slopes were computed using the individual regression equations method suggested by Fias et al. (1996). First, RTs were averaged separately for each number and each response side for every participant. Individual RT differences (dRTs) were then calculated by subtracting for each number the mean left-sided RT from the mean right-sided RT. The resulting dRTs were subsequently submitted to a regression analysis, using number magnitude as predictor variable. Unstandardized SNARC regression slopes were taken as a measure of the strength of the SNARC effect in terms of the inclination of the regression lines. Negative regression weights reflected SNARC effects in the expected direction (faster left-/right-sided RTs for small/large numbers respectively) with more negative regression slopes corresponding to stronger number-space associations.

#### Stroop Task

The English adaptation of the classical 100-item verbal paper-and-pencil version of the Stroop paradigm was used to determine Stroop-like interference control (Stroop, 1935). The task consisted of three conditions, each comprising 100 items that were displayed in a 10 × 10 matrix on an A4 sheet of paper (see **Figure 1C**). In the word reading condition (WR), participants had to read color words (“red,” “blue,” “green”) printed in black ink. In the color naming condition (CN), they named swatches of red, blue and green ink. In the interference condition (I), participants were required to indicate the color of the ink (red, blue, green) that a color word (“red,” “blue,” “green”) was written in without reading the color word (e.g., they had to indicate “red” for the color word “green” printed in red ink). Participants were instructed to name/read the different items in each condition as quickly and as accurately as possible going from left-to-right. The time needed to complete each of the three conditions was recorded in every participant using a stopwatch. The WR and CN conditions served as control conditions.

To get a single inhibitory control measure indexing each participant’s Stroop effect, we calculated RT differences between the interference and color naming conditions. This is one of the standard methods for quantifying Stroop interference control (Lansbergen et al., 2007). A greater RT difference is indicative of weaker interference control, as it reflects considerably slower RT in the interference than the color naming condition.

#### Flanker Task

The experiment was adapted from Eriksen and Eriksen (1974) and consisted of 48 trials (see **Figure 1B**). Each trial started with the display of a fixation cross (color: black; font: Arial; point size: 28) in the center of a white screen. After 400 ms, a horizontal black arrow (height: 0.69°; width: 2.06°) was presented on a white background until response or for a maximum of 1700 ms. On half of the trials, the central arrow pointed in the left direction, while on the remaining half its pointing direction was reversed. Two black horizontal flanker arrows appeared on each side of the central arrow and pointed either in the same direction than the central arrow (i.e., congruent condition, 16 trials) or in its opposite direction (i.e., incongruent condition, 16 trials). On the remaining neutral trials, the central arrow was flanked on both sides by two horizontal black bars. Participants were required to press the “A”/“L” key on a standard QWERTZ (Swiss-French) keyboard if the central arrow pointed in the left/right direction respectively. They were instructed to ignore the flanker arrows and bars. The inter-trial interval consisted of a blank screen of 500 ms. Trial sequence was identical for all participants and pseudo-randomized in a way that the correct response could not be the same more than 3 times consecutively. Moreover, the same target-distractor array did never successively appear. The actual experiment was preceded by 12 practice trials, consisting of 4 congruent, incongruent and neutral trials respectively. For each participant and every congruency condition, we computed error rates in percentages and averaged correct RTs that fell within 2.5 *SD* from the individual mean correct RT.

To incorporate error rates and RTs into a single performance measure, we computed inverse efficiency scores (IES) by dividing the means of congruent, incongruent or neutral correct RTs by their corresponding percentage accuracies for each participant (Bruyer and Brysbaert, 2011; Khng and Lee, 2014). IES thus adjusts RT performance for sacrifices in accuracy made in favor of response speed. Considering that faster responses together with fewer errors yield smaller IES, the smaller the IES is, the better the performance is.

To get a single inhibitory control measure indexing each participant’s Flanker effect, we calculated individual IES differences by subtracting congruent from incongruent IES. A greater IES difference is indicative of weaker inhibitory control, as it reflects considerably worse performance (i.e., slower RT and/or more errors) in the incongruent compared to the congruent condition.

#### Speeded Matching-to-Sample Task

The speeded matching-to-sample task was used to determine general processing speed (GPS) and described in detail by Hoffmann et al. (2014a; see also Georges et al., 2016). Each trial consisted of a centrally displayed target shape and two possible solution shapes, displayed below to the left and right. Participants had to identify the solution that was identical to the target as quickly as possible by clicking the “A”/“L” key on a QWERTZ keyboard if it appeared on the bottom left/right respectively. For each participant, we averaged correct RTs that fell within 2.5 *SD* from the individual mean correct RT.

## Results

### Descriptives

#### SNARC Effects

Split-half reliabilities were calculated for the parity and magnitude SNARC effect regression slopes using the odd–even method to control for systematic influences of practice or tiring within the tasks (see Cipora and Nuerk, 2013; Cipora et al., 2016; Georges et al., 2016, 2017; Ninaus et al., 2017). Trials were odd–even half-split (based on order of appearance) and two SNARC effect regression slopes were calculated separately for each participant and each task. The correlation coefficients were Spearman–Brown corrected to get a reliability estimate for the entire set of items. The Spearman-Brown corrected correlation coefficient was *r* = 0.56 in both the parity judgment and magnitude classification tasks.

To determine whether relatively low reliabilities could be caused by the influence of bivariate outliers, we performed linear regression analyses between odd and even SNARC effect regression slopes and identified influential data points based on the conventional Cook’s distances criterion of >4/*N* (Cook, 1979; Bollen and Jackman, 1985; see Viarouge et al., 2014 for application of this method in the SNARC context). Two separate analyses were performed – one for the parity judgment task and one for the magnitude classification task. For the parity judgment task, analysis revealed two influential data points with Cook’s distances greater than.154 (i.e., 4/26). After removal of these participants, the bivariate correlation between odd and even parity SNARC effect regression slopes remained similar (*r* = 0.35 for *N* = 24; *r* = 0.39 for *N* = 26; Fisher’s *z* for comparison of two correlations based on independent groups: *z* = 0.15; *p* = 0.88), yielding a Spearman-Brown corrected reliability estimate of *r* = 0.52. For the magnitude classification task, three influential cases were identified with Cook’s distances greater than.154 (i.e., 4/26). After removal of these three influential data points, the correlation between odd and even magnitude SNARC effect regression slopes improved from *r* = 0.39 (for *N* = 26) to *r* = 0.53 (for *N* = 23), yielding a Spearman-Brown corrected reliability estimate of *r* = 0.7. Influential cases were not removed in any of the following correlation analyses, where *N* = 26.

**FIGURE 2:**
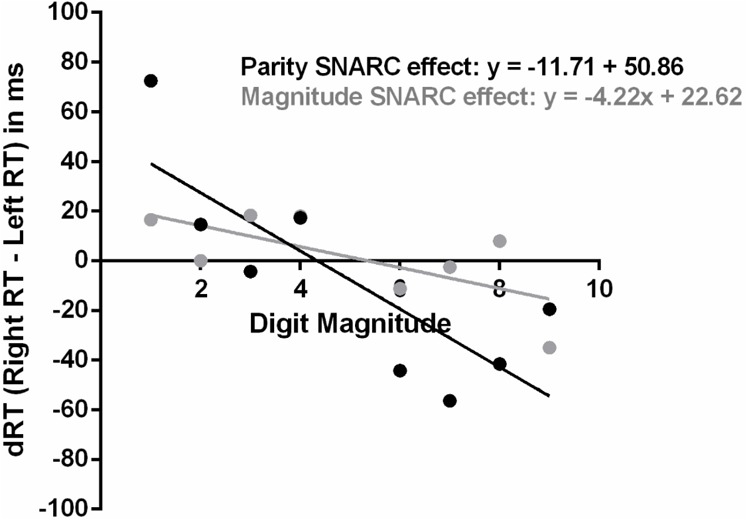
Regressions of number magnitudes onto dRTs (i.e., differences between right and left RTs) in the parity judgment and magnitude classification tasks.

The mean SNARC effect regression slope across all participants was significantly negative in the parity judgment but not the magnitude classification task [parity SNARC effect regression slope = -11.71; *SD* = 13.36; *t*(25) = -4.47; *p* < 0.001; magnitude SNARC effect regression slope = -4.22; *SD* = 12.89; *t*(25) = -1.67; *p* = 0.11; see **Figure 2**]. A repeated-measures ANOVA on the SNARC effect regression slopes also revealed a main effect of task [*F*(1,25) = 4.59; *p* = 0.042; $\eta_{p}^{2}$ = 0.16], indicating stronger number-space associations in the parity judgment than the magnitude classification task in terms of the inclination of the regression lines. Overall, a large proportion of the participants displayed a negative SNARC effect regression slope in both the parity judgment (20/26; 76.92%) and magnitude classification tasks (18/26; 69.23%).

#### Stroop Effect

The mean RTs across all participants were 43.04 s (*SD* = 8.19) in the word reading, 64.19 s (*SD* = 10.08) in the color naming and 97.04 s (*SD* = 18.96) in the interference conditions. A repeated measures ANOVA on RT including condition as within-subject variable revealed a main effect of condition [*F*(2,50) = 222.93; *p* < 0.001; $\eta_{p}^{2}$ = 0.9]. Participants performed significantly worse in the interference compared to the color naming [*t*(25) = -13.56; *p* < 0.001] and the word reading [*t*(25) = -16.13; *p* < 0.001] conditions. Performances were also significantly lower during color naming than word reading [*t*(25) = -12.51; *p* < 0.001].

The mean RT difference between the interference and color naming conditions (i.e., Stroop effect) across all participants was 32.85 s (*SD* = 12.35). Individual Stroop effects were used for the subsequent correlation analyses.

#### Flanker Effect

As for the two SNARC effects, reliability of the Flanker effect was determined using the split-half method (Greene et al., 2008; see also MacLeod et al., 2010). More concretely, congruent and incongruent trials were odd-even half-split (based on order of appearance) and Flanker effects (i.e., differences between incongruent and congruent IES) were computed separately for each half in every participant. The correlation between IES differences (i.e., Flanker effects) calculated on odd and even trials was Spearman-Brown corrected, yielding a reliability estimate of *r* = 0.63.

The mean error rates and RTs across all trials and participants were 1.2% (*SD* = 3.07) and 432 ms (*SD* = 72) in the congruent, 8.41% (*SD* = 11.17) and 490 ms (*SD* = 67) in the incongruent and 0.72% (*SD* = 2.04) and 440 ms (*SD* = 64) in the neutral conditions respectively. Error rates and RTs did not correlate in the congruent (*r* = 0.01; *p* = 0.96) and neutral (*r* = -0.23; *p* = 0.25) conditions, suggesting that these performance estimates provide different aspects of inhibitory control. Moreover, there was a speed-accuracy trade-off in the incongruent condition (*r* = -0.53; *p* = 0.006).

A repeated measures ANOVA on IES including congruency condition as within-subject variable revealed a main effect [*F*(2,50) = 47.00; *p* < 0.001; $\eta_{p}^{2}$ = 0.65]. Participants performed significantly worse on incongruent (IES = 538.69 ms; *SD* = 67.57) compared to congruent [IES = 438.09 ms; *SD* = 75.49; *t*(25) = -7.33; *p* < 0.001] and neutral [IES = 443.17 ms; *SD* = 62.69; *t*(25) = 7.13; *p* < 0.001] trials. Performances did not differ between the congruent and neutral conditions.

The mean IES difference between incongruent and congruent trials (i.e., Flanker effect) across all participants was 100.59 ms (*SD* = 69.99). Individual Flanker effects were used for the subsequent correlation analyses.

#### General Processing Speed

The mean RT across all trials and participants in the speeded matching-to-sample task was 626 ms (*SD* = 242). RTs significantly positively correlated with RTs on the parity judgment (613 ms; *SD* = 85; *r* = 0.7; *p* = < 0.001), magnitude classification (536 ms; *SD* = 74; *r* = 0.46; *p* = 0.019), Stroop (68.09 s; *SD* = 11.11; *r* = 0.4; *p* = 0.045), and Flanker tasks (454 ms; *SD* = 66; *r* = 0.41; *p* = 0.036). It thus provided a valid index of general processing speed and can be used as a control measure in a partial correlation analysis to verify whether potentially significant correlations between number-space associations and any of the interference control measures might be reduced to inter-individual differences in general processing speed.

All descriptive information is displayed in **Table 1**.

**Table 1:** Descriptive information.

Variable	All participants
Gender (f/m)	15/11
Age (years)	26.86 (*SD* = 3.29; range = 22.17 – 33.43)
Parity SNARC effect	-11.71 (*SD* = 13.36; range = -42.70 – 8.49)
Magnitude SNARC effect	-4.22 (*SD* = 12.89; range = -29.70 – 27.50)
Stroop effect (s)	32.85 (*SD* = 12.35; range = 19 – 76)
Flanker effect (ms)	100.59 (*SD* = 69.99; range = 1 – 272.56)
General processing speed (ms)	626 (*SD* = 242; range = 381 – 1372)


### Correlation Analyses

**FIGURE 3:**
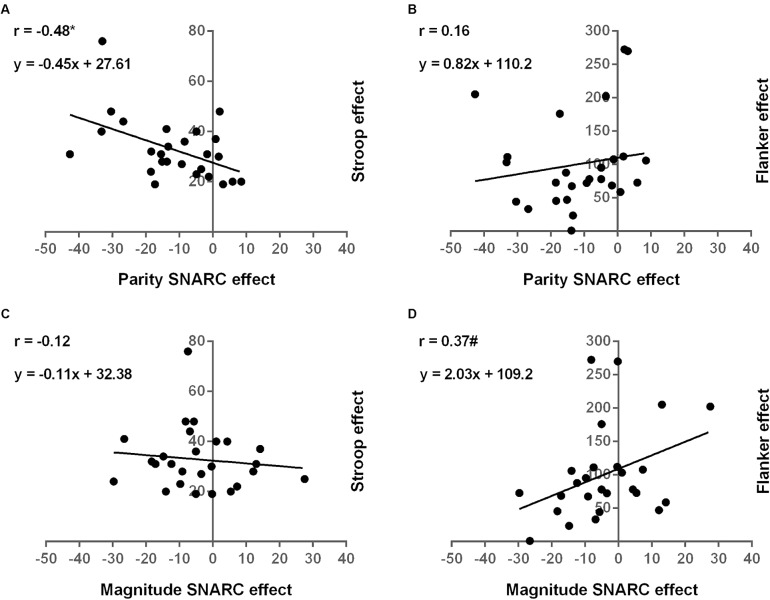
Relations between number-space associations and executive control. Correlation of the parity SNARC effect with interference control in the Stroop **(A)** and Flanker **(B)** tasks. Correlation of the magnitude SNARC effect with interference control in the Stroop **(C)** and Flanker **(D)** tasks.

All reported correlations are two-tailed, unless otherwise stated. Stronger parity SNARC effects were associated with weaker interference control in the Stroop task (*r* = -0.48; *p* = 0.012; **Figure 3A**). Conversely, no relation was observed between the parity SNARC effect and interference control in the Flanker task (*r* = 0.16; *p* = 0.44; **Figure 3B**). This difference between the relations of the parity SNARC effect with interference control in the Stroop and Flanker paradigms reached significance, as revealed by Pearson and Filon’s *z* (Pearson and Filon, 1898), assessing differences between two overlapping correlations based on dependent samples (*z* = -2.51; *p* = 0.006; one-tailed). As opposed to number-space associations in implicit tasks, stronger magnitude SNARC effects trended to be associated with better interference control in the Flanker task (*r* = 0.37; *p* = 0.06; **Figure 3D**). The magnitude SNARC effect was, however, unrelated to interference control in the Stroop task (*r* = -0.12; *p* = 0.58; **Figure 3C**). The difference between the correlations of the magnitude SNARC effect with Stroop and Flanker effects was significant (*z* = -1.80; *p* = 0.04; one-tailed). In line with previous findings, the parity and magnitude SNARC effects did not correlate (*r* = 0.08; *p* = 0.7). The difference between the relations of the Stroop effect with the parity and magnitude SNARC effects, however, only trended toward significance (*z* = -1.54; *p* = 0.06; one-tailed). Likewise, no significant difference could be observed between the correlations of the SNARC effects in implicit and explicit tasks with the Flanker effect (*z* = -0.87; *p* = 0.19; one-tailed). Performances on the Stroop and Flanker tasks did also not correlate (*r* = -0.14; *p* = 0.5), confirming qualitative differences between these interference measures. Finally, general processing speed did not relate to any of the SNARC effects or inhibitory control measures (all *p*s > 0.05).

Considering the non-perfect reliabilities of the SNARC effect regression slopes, we corrected bivariate correlations for attenuation using Spearman’s correction for attenuation formula, corresponding to r_xy_ /sqrt(r_xx_^∗^r_yy_), with r_xx_ and r_yy_ coding for the reliabilities of X and Y respectively (Spearman, 1904, 1910; Muchinsky, 1996; see also Cipora and Nuerk, 2013; Gloria et al., 2016; Georges et al., 2017, for a comparable application of this correction for attenuation method). This procedure determines the correlation between two variables if they were perfectly reliable, and therefore provides for a more accurate estimate of the correlation between two parameters. Attenuated and disattenuated correlation coefficients are shown in the upper and lower part of **Table 2** respectively.

**Table 2:** Correlation analysis.

	1	2	3	4	5
(1) Parity SNARC effect	–	**0.08**	**-0.48^∗^**	**0.16**	**-0.12**
(2) Magnitude SNARC effect	0.14	–	**-0.12**	**0.37#**	**-0.28**
(3) Stroop effect	-0.64	-0.16	–	**-0.14**	**0.04**
(4) Flanker effect	0.27	0.62	-0.18	–	**-0.22**
(5) General processing speed	-0.16	-0.37		-0.28	–


*Attenuated correlation coefficients are displayed in bold in the upper part of the table. Disattenuated correlation coefficients are displayed in the lower part of the table. ^∗^*p* < 0.05; #*p* = 0.06.*

All the above relations remained similar when controlling for general processing speed in a partial correlation analysis (see **Table 3**).

**Table 3:** Partial correlation analysis controlling for general processing speed.

	1	2	3	4
(1) Parity SNARC effect	–	0.05	-0.48^∗^	0.14
(2) Magnitude SNARC effect		–	-0.11	0.33
(3) Stroop effect			–	-0.13
(4) Flanker effect				–


*^∗^*p* < 0.05.*

## Discussion

### Inter-Individual Differences in Number-Space Associations During Parity Judgments Relate to Interference Control in the Stroop Task

Stronger number-space associations in the parity judgment task correlated with weaker interference control in the Stroop task in young adults with diagnosed or self-reported ADHD. This relation remained significant even after controlling for general processing speed, previously implicated in both the parity SNARC (e.g., Wood et al., 2008; Cipora and Nuerk, 2013; Hoffmann et al., 2014b) and Stroop effects (e.g., Bugg et al., 2007; Hoffmann et al., 2014b). The present findings extend the recently reported relation between stronger parity SNARC effects and weaker Stroop inhibitory control in the elderly and confirm the hypothesis that the null relation in young healthy participants can be explained by their near ceiling performances on the Stroop task (Hoffmann et al., 2014b).

In contrast, number-space associations during parity judgments were not related to interference control in the Flanker task in the present population. It is unlikely that this null relation can be explained by insufficient variance in the Flanker effect due to near ceiling task performances, considering the tendency for a positive relation between interference control in the Flanker task and number-space associations in the magnitude classification task (discussed in the next section). Moreover, individuals with ADHD were previously shown to feature abnormal inhibitory control in both the Stroop (Nigg et al., 2005; Walker et al., 2000; King et al., 2007) and Flanker paradigms (Lundervold et al., 2011). The spatial coding mechanisms underlying the parity SNARC effect thus depend on those inhibitory control processes indexed by the Stroop but not the Flanker effect. Overall, this provides valuable information regarding the type of conflict encountered during parity judgments, thereby advancing our understanding of the spatial coding processes underlying the parity SNARC effect.

To characterize the coding mechanisms accounting for the parity SNARC effect, it is important to firstly understand the cognitive processes underlying interference control in the Stroop and Flanker tasks. Interference in the Stroop paradigm originates at the semantic level from an attribute that is intrinsic to the target stimulus (i.e., the meaning of the color word conflicts with the semantic representation of the ink color, e.g., Klein, 1964; La Heij, 1988). Moreover, the distracting color word meaning is highly salient, considering that literate individuals are primed to automatically access a word’s meaning upon sight prior to processing any additional features (Ashcraft and Radvansky, 2010). Conversely, interference in the Flanker paradigm occurs spatially instead of semantically from lateral arrows that are drawn from the same set of stimuli than the target stimulus (Eriksen and Schultz, 1979). The relation between the parity SNARC and Stroop (but not Flanker) effects thus suggests that the spatial code associated with numerical magnitude during parity judgments is semantic in nature and/or intrinsic to the target stimulus (see **Table 4**). Since the Stroop as opposed to the Flanker task yields basically no perceptual interference (Valle-Inclán, 1996), the conflict in the parity judgment task is also unlikely of perceptual nature. This outcome is in line with the parity judgment paradigm, where the task-relevant parity status and the conflicting spatial code associated with the automatically activated yet task-irrelevant magnitude information reflect distinct semantic properties of the same target number.

**Table 4:** Characteristics of the spatial code associated with numerical magnitude during parity judgments and magnitude classifications.

		Parity judgment	Magnitude classification	
			
			*Alternative a*	*Alternative b*
**Spatial code**	**Relevance**	Irrelevant	Irrelevant	Relevant
**characteristics**	**Nature**	Verbal	Visual	Visual
	**Origin**	Intrinsic to target number	Extrinsic to target number	Intrinsic to target number
	**Processing stage**	Response selection	Encoding	Encoding
	**Processing mechanism**	Suppression via biasing units reflecting relevant response	Suppression via spatial filtering	Activation via selective attention


*In the parity judgment task, the irrelevant verbal-spatial code associated with the numerical magnitude of the target number interferes with the spatial location of the response based on parity status during response selection. Interference is resolved via biasing units coding the spatial location of the relevant response. For the magnitude classification task, two alternatives (a and b) are outlined. According to alternative (a), the irrelevant visuospatial codes associated with the numerical magnitudes of the numbers represented adjacently to the target number on the MNL interfere with the processing of the target number during encoding. Interference is resolved via the spatial filtering of the irrelevant numerical magnitude representations on the MNL. According to alternative (b), the relevant visuospatial code associated with the numerical magnitude of the target number is activated during encoding via selective attention.*

While distraction in the Flanker task is provided by externally available visuospatial information (i.e., the flanking arrows), the distracting color word meaning in the Stroop paradigm is rather verbal in nature. The Stroop task is highly left lateralized, most prominently in the left dorsolateral prefrontal cortex and inferior frontal areas, previously implicated in the resolution of verbal conflict (Jonides et al., 1998; Leung et al., 2000; Jonides and Nee, 2006). In the present Stroop paradigm, responses were also given verbally, thereby adding to the already rather verbal nature of the Stroop task. The strong relation between the parity SNARC and Stroop effects thus suggests that the distracting spatial code associated with numerical magnitude in the parity judgment task might also be verbal in nature (see **Table 4**). In line with previous claims, this suggests that the parity SNARC effect predominantly results from verbal-spatial polarity coding as opposed to arising from the spatial coding of numerical magnitudes on a horizontally oriented MNL (Gevers et al., 2010; Georges et al., 2017).

According to the dimensional overlap model by Kornblum et al. (1990; see also Kornblum and Lee, 1995; Zhang et al., 1999), interference in the Flanker task mainly reflects a stimulus-stimulus conflict, where the pointing directions of the task-irrelevant flanking arrows interfere with that of the targeted central arrow at the early stage of stimulus encoding. Such interference is likely resolved via the spatial filtering of the perceptual distractors and the narrowing of the attentional focus to the task-relevant central arrow location (Wendt et al., 2012). Conversely, conflict in the Stroop paradigm occurs at multiple stages of stimulus processing (Zhang and Kornblum, 1998; Milham et al., 2001; De Houwer, 2003). In addition to the semantic stimulus-stimulus conflict at earlier processing stages (e.g., Klein, 1964; Kornblum et al., 1990; Sharma and McKenna, 1998; Schmidt and Cheesman, 2005; Goldfarb and Henik, 2007), stimulus-response conflict arises during response selection (e.g., Cohen et al., 1990; MacLeod, 1991; van Veen and Carter, 2005; Szucs and Soltész, 2010), when the task-relevant ink color and the irrelevant meaning of the color word activate competing responses. Such stimulus-response conflict is then probably resolved via biasing units reflecting the task-relevant semantic dimension (i.e., the ink color of the color word; Szucs et al., 2009). The relation between number-space associations in the parity judgment task and interference control in the Stroop paradigm thus suggests that the parity SNARC effect also mainly originates at later processing stages during response selection (see **Table 4**). Accordingly, the response provoked by the task-irrelevant numerical magnitude-associated spatial code competes/conflicts with that induced by the task-relevant parity status prior to response execution. Such competition is likely resolved via biasing units coding the response associated with the task-relevant parity status (see **Table 4**). Considering the absence of a relation between the parity SNARC and Flanker effects, interference in the parity judgment task is unlikely controlled by filtering mechanisms already at the early stage of number encoding. This outcome is in line with previous models proposed to account for the parity SNARC effect (Keus et al., 2005; Gevers et al., 2006). According to Gevers et al. (2006), the parity SNARC effect results from the interference of two processing routes operating in parallel. The conditional route links task-relevant parity information with response keys based on task instructions, while the unconditional route conveys the automatic association between numerical magnitude and space. On congruent trials, both routes activate the same response location, while on incongruent trials responses are slowed down and more error-prone since the two routes activate competing outcomes.

Evidence for such parallel processing of task-relevant and irrelevant information and of conflict resolution mainly at the response selection stage during parity judgments has also been provided by EEG studies. Namely, congruency effects were previously reported on the latency of the lateralized readiness potential (Keus et al., 2005; Gevers et al., 2006), an EEG component considered to be the output of response selection stages (Gratton et al., 1988; Coles, 1989; for a review, see also Leuthold et al., 2004). In addition and in line with observations regarding the Stroop effect (Ilan and Polich, 1999; Zurrón et al., 2009; for a review, see Sahinoglu and Dogan, 2016), the P300 peak latency did not show an onset difference between congruent and incongruent trials in the parity judgment task (Gevers et al., 2006), indicating that the conflict indexed by the parity SNARC effect is unlikely detected at early perceptual stages.

The assumption that the conflict indexed by the parity SNARC effect originates at later processing stages during response selection also agrees with findings regarding stronger parity SNARC effects in the elderly compared to young healthy individuals (Hoffmann et al., 2014b; Ninaus et al., 2017). Elderly persons featured weaker interference control in the Stroop paradigm than young controls (West and Alain, 2000; Van der Elst et al., 2006), suggesting an age-associated decline in conflict resolution particularly at later response selection stages. In contrast, the resolution of stimulus-stimulus conflict at earlier processing stages in the Flanker task did not differ between younger and older participants as reflected by similar behavioral performances of both age groups (Falkenstein et al., 2001; Nieuwenhuis et al., 2002).

### Inter-Individual Differences in Number-Space Associations During Magnitude Classifications Relate to Interference Control in the Flanker Task

Inter-individual variability in the strength of number-space associations during explicit magnitude classifications did not relate to inter-individual differences in the Stroop effect. Conversely, stronger magnitude SNARC effects were associated with better interference control in the Flanker task. However, it should be noted that this correlation did not reach significance, also not prior to partialling out the effects of general processing speed. Nonetheless, the relation between more pronounced number-space associations during explicit magnitude classifications and better interference control in the Flanker paradigm was significantly different from the null correlation between the magnitude SNARC and Stroop effects.

The latter null relation might suggest that the spatial code associated with numerical magnitude during explicit classifications is not of verbal nature, akin to the verbal interference encountered in the Stroop paradigm (Jonides et al., 1998; Leung et al., 2000; Jonides and Nee, 2006) and probably also during parity judgments. This lines up with previous findings indicating that the magnitude SNARC effect was selectively abolished by a visuospatial but not verbal WM load, highlighting the importance of visuospatial coding mechanisms (van Dijck et al., 2009). Moreover, Georges et al. (2017) reported a relation between stronger magnitude SNARC effects and greater preferences for spatial as opposed to object visualization. Number-space associations during explicit magnitude classifications thus likely predominantly depend on visuospatial processing resources in the right parietal cortex associated with spatial visualization (Lamm et al., 1999; see **Table 4**). The absence of a correlation between number-space associations in the magnitude classification task and interference control in the Stroop paradigm might also indicate that the magnitude SNARC effect differs from conflict that originates from a semantic feature intrinsic to the target stimulus (i.e., the central number). Furthermore, interference in the magnitude classification task might diverge from conflict that is mainly resolved at the response selection stage, such as the conflict induced by the irrelevant color word meaning in the Stroop paradigm. The null relation between the magnitude SNARC and Stroop effects could, however, also simply suggest that no conflict arises from the spatial code associated with numerical magnitude during explicit classifications.

When considering the tendency for an association between the magnitude SNARC and Flanker effects, it might suggest that the potential interference during explicit classifications originates from irrelevant visuospatial information extrinsic to the target stimulus (see **Table 4**, alternative a). Additionally, it could indicate conflict resolution directly at the early stage of stimulus encoding via spatial filtering (see **Table 4**, alternative a). At first, this idea seems difficult to reconcile with the magnitude classification paradigm, considering that it only comprises a single task-relevant centrally displayed number. If extrinsic distraction might be encountered during magnitude classifications, it can only originate internally. One possibility is for instance that interference arises from task-irrelevant numerical magnitudes represented adjacently to the target number on a horizontal MNL (or sequence within WM; see e.g., Fias et al., 2011). Indirect support for such an interplay between the externally available task-relevant number and internally represented task-irrelevant numerical magnitudes was provided by Nuerk et al. (2005). Their findings suggested that the representation of closely related task-irrelevant numbers can interfere with task-relevant numerical magnitude classifications at least when these distracting numbers are externally available. Of course, the assumption of such interference by internally represented task-irrelevant numerical magnitudes is only valid if the spatial code associated with numerical magnitude during explicit classifications is indeed visual instead of verbal in nature. A greater ability to suppress such task-irrelevant spatial-numerical activations at earlier processing stages (akin to the spatial filtering of distractors in the Flanker task) might then facilitate the processing of the task-relevant numerical magnitude together with its associated spatial code, manifesting in stronger magnitude SNARC effects. This explanation could then account for the positive relation between stronger magnitude SNARC effects and better interference control in the Flanker task.

Alternatively, the trend for a relation between stronger magnitude SNARC effects and better inhibitory control in the Flanker task might indicate that a greater ability to selectively focus attention on task-relevant information (as indexed by better interference control in the Flanker task; see Wendt et al., 2012) is associated with stronger number-space associations during explicit magnitude classifications. Of course, this entails that the spatial code associated with the task-relevant numerical magnitude is also relevant rather than distracting for successful resolution of the magnitude classification task (see **Table 4**, alternative b). The relevance of spatial-numerical mappings during explicit magnitude classifications could then also account for the lack of a correlation between the magnitude SNARC and Stroop effects. Moreover, it seems likely considering that coding small/large numerical magnitudes as left/right on the MNL (or within WM) might assist left-/right-sided numerical magnitude classifications. It would also provide an explanation for the observation that stronger magnitude SNARC effects are not related to weaker arithmetic performances (Georges et al., 2017), contrary to the parity SNARC effect (e.g., Cipora et al., 2016; Georges et al., 2017; but see Cipora and Nuerk, 2013). In general, more linear spatial representations of numerical magnitudes, as assessed using number line estimations, are commonly associated with better magnitude comparison performances (Laski and Siegler, 2007) as well as higher math skills (Link et al., 2014). These findings thus highlight the importance/relevance of spatial-numerical representations for arithmetic performances.

### Intra-Individual Differences in Number-Space Associations and Task-Dependent Differences in the Relation to Interference Control

The present results provide further evidence for the previously reported intra-individual variability in number-space associations depending on the implicit or explicit nature of numerical magnitude processing (van Dijck et al., 2009; Georges et al., 2017). More concretely, parity and magnitude SNARC effects were uncorrelated and related (or at least tended to relate) inversely to distinct inhibitory control measures, namely negatively with the Stroop and positively with the Flanker effects respectively. This heterogeneity in the cognitive processes underlying the SNARC effect generally agrees with studies indicating that both long-term spatial coding mechanisms such as the spatial representation of numerical magnitudes on a MNL and temporary associations between the ordinal position of numerical magnitudes and space in WM might exist in parallel (Ginsburg and Gevers, 2015; Huber et al., 2016; but see Abrahamse et al., 2016).

Previous explanations for such intra-individual variations in number-space associations depending on the number processing task suggested task-related differences in the nature of the numerical magnitude-associated spatial code, with verbal- and visuospatial coding processes probably underlying the parity and magnitude SNARC effects respectively (van Dijck et al., 2009; Gevers et al., 2010; Georges et al., 2017). This assumption might further be supported by the present findings. Namely, only the parity SNARC effect correlated with interference control in the Stroop paradigm, reflecting the suppression of task-irrelevant verbal information (i.e., the color word meaning).

The current results, however, allow for an additional (or even alternative) explanation regarding intra-individual differences in number-space associations depending on the number processing task. Namely, as already discussed above, the relations of the parity and magnitude SNARC effects with stimulus–response and stimulus–stimulus conflict resolution in the Stroop and Flanker paradigms respectively suggests that the task-dependency of number-space associations might result from task-related differences in the processing stages of the spatial code associated with numerical magnitude, irrespective of its visual or verbal nature. While the conflict provided by the numerical magnitude-associated spatial code during parity judgments might predominantly be resolved at the response selection stage via biasing units coding the task-relevant response location (see **Table 4**), the potential conflict during explicit magnitude classifications probably rather originates from extrinsic distractors and is resolved via their spatial filtering at earlier processing stages (see **Table 4**, alternative a). The conflicts indexed by the parity and magnitude SNARC effects would thus have distinct origins and be resolved via different mechanisms at different processing stages, thereby potentially explaining the task-dependency of number-space associations.

Alternatively, as already mentioned before, differences in the relevance of the spatial code associated with numerical magnitude during parity judgments and magnitude classifications and consequently in its processing (inhibition vs. activation respectively) could probably underlie the task-dependency of number-space associations (see **Table 4**).

### Limitations and Future Directions

First, it should be reminded that split-half reliabilities for both the parity and magnitude SNARC effect regression slopes were relatively low. Lower reliabilities are, however, not unusual in SNARC-related studies. Comparably low reliabilities were also reported in previous studies by means of both internal consistency (Cipora and Nuerk, 2013; Viarouge et al., 2014; Georges et al., 2016, 2017; Cipora et al., 2018) as well as test–retest stability (Viarouge et al., 2014).

To increase reliability estimates, the length of the parity judgment task was increased, which was shown to yield better split-half reliability estimates (Cipora and Wood, 2012, 2017; see also Cipora and Nuerk, 2013; Cipora et al., 2016). Nonetheless, the Spearman–Brown corrected correlation coefficient in the present parity judgment task was comparable to that in Georges et al. (2017) using a task that included only half of the number of trials. Moreover, similar split-half reliability estimates were obtained for the parity and magnitude SNARC effects, albeit the parity judgment task had twice the length of the magnitude classification paradigm. Increasing the number of repetitions per stimulus in the parity judgment task did thus not seem to enhance split-half reliability in the current study. It should, however, be noted that the present study only included individuals with diagnosed or self-reported ADHD, generally featuring relatively high intra-individual variability in RTs (Castellanos et al., 2005; Vaurio et al., 2009). This might thus have generally accounted for the lower reliabilities, despite the increase in some of the task lengths.

Importantly, the relatively poor reliabilities of the parity and magnitude SNARC effect regression slopes could have negatively impacted the correlations reported in the current study. Namely, the upper bound of a correlation between two parameters depends on their reliabilities in that the highest correlation between two variables equals the square root of the product of their reliabilities [i.e., sqrt(r_xx_^∗^r_yy_), with r_xx_ and r_yy_ coding for the reliabilities of X and Y respectively]. The correlation between two variables is thus weakened by measurement error, such that true correlations between measures with poor reliability might be overlooked (Osborne and Waters, 2002, p. 2). Consequently, we need to be careful when drawing conclusions about (the absence of) relations between number-space associations and interference control from the present findings. Nevertheless, any task-related differences in the relations between number-space associations and the different interference control measures cannot be ascribed to low measurement reliability, since split-half reliability estimates for the parity and magnitude SNARC effect regression slopes were equally low.

Another drawback of the present study could be the relatively small sample size of *N* = 26. A *post hoc* power analysis based on effect size, conventional alpha level, and sample size (i.e., *N* = 26) using the program G^∗^Power (Faul et al., 2007, 2009) revealed that the probability of rejecting a false null hypothesis was 81% for large (*r* = 0.5), 34% for medium (*r* = 0.3) and 8% for small (*r* = 0.1) effect sizes. The present study had thus sufficient power to detect a significant relation between the SNARC effect and inhibitory control at the large effect size level. Conversely, less than adequate statistical power was obtained at the small to medium effect size level to reject an incorrect null hypothesis. The lack of sufficient power for detecting small to medium effect sizes could potentially account for the non-significant relation between stronger number-space associations during magnitude classifications and better interference control in the Flanker task in the current sample.

Future studies should also consider the inclusion of control variables. Especially the involvement of verbal and visuospatial WM could be assessed in greater detail. Relations between number-space associations and inhibitory control might indeed be (partially) confounded by WM processes. WM is not only implicated in the Stroop (Long and Prat, 2002; Kane and Engle, 2003; Hutchison, 2011) as well as Flanker effects (Redick and Engle, 2006; Heitz and Engle, 2007), but also likely contributes to number-space associations (van Dijck et al., 2009). Nonetheless, it should be noted that Hoffmann et al. (2014b) controlled for the influence of verbal WM in their study, thereby excluding the possibility that the relation between stronger parity SNARC effects and weaker interference control in the Stroop paradigm might be confounded by verbal WM.

Future research could also elaborate on the assumption that no interference originates from the spatial code associated with numerical magnitude during explicit classifications by investigating whether the strength of the magnitude SNARC effect varies with age, similarly to the age-associated increase in number-space associations during parity judgments (Hoffmann et al., 2014b; Ninaus et al., 2017). Inhibitory control declines with age (see Glisky, 2007) mostly regarding conflict resolution at later response selection stages (Falkenstein et al., 2001; Nieuwenhuis et al., 2002; Van der Elst et al., 2006), while target selection processes usually remain intact even in the elderly (West and Alain, 2000). Consequently, if the magnitude SNARC effect indeed does not index interference control, its strength should not be altered by aging.

## Conclusion

Stronger parity SNARC effects were associated with weaker interference control in the Stroop but not Flanker task in young adults with diagnosed or self-reported ADHD. Number-space associations in the parity judgment task thus index conflict resolution akin to the Stroop effect. In other terms, the parity SNARC effect likely reflects interference between the (probably) verbal-spatial code associated with numerical magnitude and the spatial location of the response associated with parity status at later processing stages during response selection (see **Table 4**). Conversely, the magnitude SNARC effect was not related to interference control in the Stroop paradigm. Stronger number-space associations during explicit magnitude classifications, however, tended to be associated with better conflict resolution in the Flanker task. The (probably) visuospatial code associated with numerical magnitude is thus likely relevant during explicit magnitude classifications, with its activation at the early stage of stimulus encoding underlying the magnitude SNARC effect (see **Table 4**, alternative b). Overall, the present findings suggest that the relevance/importance of number-space associations for numerical judgments depends on the implicit or explicit nature of the number processing task. While the spatial code associated with numerical magnitude seems to assist explicit magnitude classifications (and is therefore activated at the encoding stage), it seems to interfere with parity judgments (and is therefore suppressed at the response selection stage). Such differences in the relevance of the numerical magnitude-associated spatial code during parity judgments and magnitude classifications and in the related executive control mechanisms monitoring its processing (suppression vs. activation respectively) might account for the previously reported task-dependency of number-space associations.

## Author Contributions

CG, DH, and CS: conceived and designed the experiments. CG: analyzed the data. CG, DH, and CS: wrote the paper.

## Conflict of Interest Statement

The authors declare that the research was conducted in the absence of any commercial or financial relationships that could be construed as a potential conflict of interest.

## Footnotes

1 Since previous research indicated that split-half reliability was significantly lower for the parity than the magnitude SNARC effect (Georges et al., 2017), we decided to double the number of trials in the parity judgment task, as this was suggested to considerably enhance reliability estimates (Cipora and Wood, 2012; see also Cipora and Nuerk, 2013; Cipora et al., 2016). Due to time constraints, we did, however, not increase the length of the magnitude classification task.
